# The Beneficial Effects of Essential Oils in Anti-Obesity Treatment

**DOI:** 10.3390/ijms222111832

**Published:** 2021-10-31

**Authors:** Anna De Blasio, Antonella D’Anneo, Marianna Lauricella, Sonia Emanuele, Michela Giuliano, Giovanni Pratelli, Giuseppe Calvaruso, Daniela Carlisi

**Affiliations:** 1 Laboratory of Biochemistry, Department of Biological, Chemical and Pharmaceutical Sciences and Technologies (STEBICEF), University of Palermo, 90127 Palermo, Italy; anna.deblasio@unipa.it (A.D.B.); antonella.danneo@unipa.it (A.D.); michela.giuliano@unipa.it (M.G.); giuseppe.calvaruso@unipa.it (G.C.); 2 Institute of Biochemistry, Department of Biomedicine, Neurosciences and Advanced Diagnostics (BIND), University of Palermo, 90127 Palermo, Italy; sonia.emanuele@unipa.it (S.E.); giovanni.pratelli@unipa.it (G.P.)

**Keywords:** obesity, metabolic syndrome, essential oils

## Abstract

Obesity is a complex disease caused by an excessive amount of body fat. Obesity is a medical problem and represents an important risk factor for the development of serious diseases such as insulin resistance, type 2 diabetes, cardiovascular disease, and some types of cancer. Not to be overlooked are the psychological issues that, in obese subjects, turn into very serious pathologies, such as depression, phobias, anxiety, and lack of self-esteem. In addition to modifying one’s lifestyle, the reduction of body mass can be promoted by different natural compounds such as essential oils (EOs). EOs are mixtures of aromatic substances produced by many plants, particularly in medicinal and aromatic ones. They are odorous and volatile and contain a mixture of terpenes, alcohols, aldehydes, ketones, and esters. Thanks to the characteristics of the various chemical components present in them, EOs are used in the food, cosmetic, and pharmaceutical fields. Indeed, it has been shown that EOs possess great antibiotic, anti-inflammatory, and antitumor powers. Emerging results also demonstrate the anti-obesity effects of EOs. We have examined the main data obtained in experimental studies and, in this review, we summarize the effect of EOs in obesity and obesity-related metabolic diseases.

## 1. Introduction

Obesity is a condition characterized by an excessive accumulation of body fat resulting from genetic, psychological, and socio-environmental factors that leads to an imbalance between calorie intake and energy expenditure in favor to the former [1,2,3,4]. As established by the World Health Organization (WHO), the term “obesity” is used when the value of the Body Mass Index (BMI, calculated by dividing the weight expressed in kilograms by the square of the height expressed in meters) is greater than 30 [5]. Obesity is one of the main problems concerning public health due to its constant increase, particularly in Western countries [6]. Obesity is, in fact, a known risk factor for serious chronic diseases such as type 2 diabetes, cardiovascular and respiratory diseases, tumors, and psychological disorders [1]. According to the data made public by WHO, worldwide obesity has nearly tripled since 1975. In 2016, more than 1.9 billion adults, 18 years and older, were overweight, of which over 650 million were obese. A worrying fact is that 38 million children under the age of 5 and over 340 million children and adolescents aged 5–19 were overweight or obese.

Recent studies, performed both with in vivo and in vitro systems, show that essential oils (EOs) exert a broad-spectrum therapeutic potential against obesity and its related-diseases [7,8].

**Figure 1:**
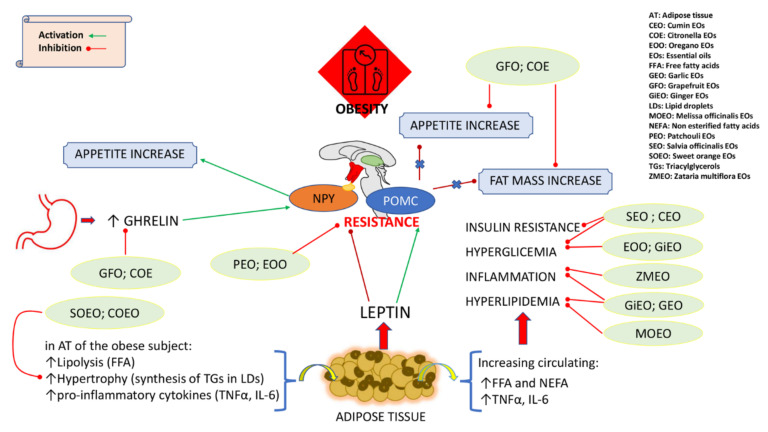
Schematic representation of effects of EOs in reducing/preventing obesity and in obesity-related diseases.

The purpose of this review is to summarize the effects of EOs in reducing/preventing obesity or in obesity-related diseases, such as metabolic syndrome (Figure 1). We will also highlight the effect of EOs on the microbiota, considering that the dysbiosis can contribute to a pathological state associated with obesity [9,10,11].

## 2. Essential Oils (EOs)

Essential oils (EOs) are present in flower petals, exocarp, resin, tree bark, and the roots of aromatic and medicinal plants. Known also as “essences”, they are characterized by the presence of volatile substances at room temperature, which can give them different smells and fragrances. It is no coincidence, therefore, that EOs are also known as “volatile oils” and that their components are defined as “aromatic” (hence the term aromatic plants). The synthesis and accumulation of these essences takes place within different secretory structures in the various plant families such as secretory cavities in Myrtaceae and Rutaceae, glandular trichomes in Lamiaceae, and resin ducts in Asteraceae and Apiaceae [12].

EOs are a heterogeneous blend of numerous chemical compounds produced by the secondary metabolism of plants and are often responsible for the distinctive odors of plants. They play an important role in the protection of plants thanks to their antibacterial, antiviral, antifungal, and insecticidal action and can also act as an attraction towards pollen insects to favor the dispersion of seeds and pollen. EOs typically consist of 20–60 different compounds of which two or three represent 20–70% of the essence while the others are present only in traces [13]. The main components of EOs are monoterpenes and sesquiterpenes; they also contain, in a minor extent, aromatic compounds derived from phenylpropane. All components have a low molecular weight and for this reason they are liquid at room temperature [14]. Monoterpenes can be linear or cyclic compounds through redox reactions, and monoterpenes can generate other compounds with functional groups typical of alcohols, aldehydes, ketones, esters, and ethers [13].

Even sesquiterpenes can be distinguished into linear and cyclical ones and can undergo redox reactions generating functional groups. Aromatic compounds derived from phenylpropane may be present to a lesser extent in EOs [15].

Among the main bioactive components of EOs we can find:

– Carvacrol (2-methyl-5-[1-methylethyl] phenol), which is the main product of numerous aromatic plants including Origanum, Thymus, Satureja, and Thymbra. Several studies show that carvacrol has antimicrobial, anti-fungal, anti-inflammatory, antioxidant, and antiproliferative activities [16].
– Limonene, which is the main constituent of EOs extracted from Citrus, but is also present in the resin of conifers, particularly Pinaceae. It has anti-inflammatory, antioxidant, and anticancer properties. It is commonly used as a natural food flavoring [17].
– Trans-anethole (trans-1-methoxy-4-propenyl-benzene), which is the main component of EOs extracted from more than 20 species including fennel, anise, and star anise. It can have anti-inflammatory, anticancer and antidiabetic effects. It is used as a natural food flavoring [18].
– Cinnamaldehyde (trans-cinnamic aldehyde), which is known to be the main component of cinnamon flavor. Several studies have highlighted the anti-inflammatory activity of this aldehyde; moreover, it has the following properties: anti-infective (antibacterial, antifungal, antiviral), antiseptic, mucolytic and expectorant, analgesic, and anti-edematous. It is commonly used as a natural food flavoring [19].

EOs are obtained by the steam distillation, hydrodistillation, dry distillation, or cold pressing of plant organs [14]. The classic method of extraction is based on current-distillation of steam. Essences can be extracted from fruits (e.g., citrus) by cold pressing the exocarps of the fruit [15]. A variant of steam distillation is hydrodistillation, in which the plant material is immersed in water that is heated to a boil. It is generated in this case by a stream of steam that carries the essential oil into the condenser and then into the decanting system. More modern methods involve the use of microwaves and fluids such as supercritical carbon dioxide, which is in an intermediate stage between gaseous and liquid and has a high solvating capacity [20].

## 3. Adipose Tissue and Obesity

The adipose tissue (AT) is a heterogeneous tissue composed by adipocytes and non-adipocyte cellular components including inflammatory cells (macrophages), immune cells, and fibroblasts. Mainly two types of adipose tissue exist in mammals: the white adipose tissue (WAT) and the brown adipose tissue (BAT). WAT stores energy as triacylglycerols (TGs), while BAT is involved in the maintenance of body temperature by promoting thermogenesis [21]. Especially after birth and in the prepubertal period, adipose tissue grows mainly due to an increase in the number of adipocytes (hyperplasia). The proliferation rate of adipocytes decreases during adolescence and remains stable during adulthood, when the adipose tissue initially expands with an increase in the size of adipocytes (hypertrophy). During periods of positive energy balance, such as overeating or a sedentary lifestyle, the expansion of adipose tissue can be achieved by hyperplasia and/ or hypertrophy, leading to obesity [22,23].

WAT is more abundant and is found in subcutaneous tissue (panniculus adipose), in the empty viscera of the abdominal cavity or mediastinum, and in different muscle groups [24]. White adipocytes contain a single lipid droplet that in mature cell is so large that it displaces the nucleus and remaining cytoplasm to the cell periphery. In humans, WAT has an energy reserve function, synthesizing and storing TGs [25]. TGs accumulate in adipose tissue in “lipid droplets” (LDs), outside of which is perilipin, a protein that regulates their organization and inhibits lipolysis [26]. At least five perilipin classes have been identified encoded by mRNA splice variants of a single gene [27]. Other roles of the WAT are to act as a thermal insulator (subcutaneous adipose panniculus); act as a mechanical shock absorber in areas particularly subject to pressure; and allow muscle bundles to slide over each other without compromising their functional integrity. Notably, WAT is also an endocrine organ which produces biologically active substances called adipokines, including leptin, adiponectin, complement components, plasminogen activator inhibitor-1 (PAI-1), proteins of the renin-angiotensin system, resistin, and pro- and anti-inflammatory cytokines [28,29,30]. Adipokines, especially those produced by visceral WAT, seem to represent the biochemical link between obesity, inflammation, and metabolic syndrome. In fact, the WAT present in muscle tissue is capable of secreting free fatty acids (FFAs), interleukin 6 (IL-6), and tumor necrosis factor α (TNFα). These factors, released at high levels by an hypertrophic WAT, play a role in the development of insulin resistance and type 2 diabetes [31]. Moreover, WAT associated with the heart muscle secretes numerous cytokines resulting in local inflammatory events that can contribute to the development of atherosclerosis and hypertension [32,33].

The adipocytes that make up the BAT are smaller than those found in the WAT. They contain many small LDs and numerous and large mitochondria (from which the coloring derives). BAT is highly represented in infants and progressively decreases with age, remaining metabolically active. It is generally localized in the interscapular region, in the axillary, along the great blood vessels, and around the kidney and adrenal gland [34]. The function of BAT is to produce heat (thermogenesis) [35], due to the presence in the mitochondria of the uncoupling protein-1 (UCP-1), or thermogenin, which allows the generation of heat through the oxidation of fatty acids (FAs) [36]. It is interesting to note that the metabolic activity of BAT is inversely correlated with the body fat mass index [34]. Indeed, the activation of BAT by the repeated exposure to cold increases thermogenesis and reduces fat mass, suggesting that the activation of this process in humans can reduce obesity [35,37].

A third type of adipocytes, called beige, has been recently discovered, located within the WAT especially at the subcutaneous level. Beige adipocytes arise from white adipocytes under a process known as browning [38]. The beige adipocytes biogenesis in WAT can be promoted by different signals such as cold, exercise, and adrenergic receptors. Beige adipocytes have features midway between white and brown. Similarly to brown adipocytes, beige cells also possess numerous LDs and mitochondria that express UCP-1 and are capable of activating thermogenesis [39,40,41]. Precisely for these intermediate characteristics, it has been defined as beige [38].

### 3.1. Regulation of Lipogenesis and Lipolysis

The accumulation of fat in AT is determined by the balance between the synthesis of TGs (lipogenesis) and their hydrolysis (lipolysis). The balance between the accumulation and mobilization of TGs in AT is mainly under the control of numerous hormones, tissue innervation, and blood flow.

The lipostatic theory explains the relative constancy of body weight based on a negative feedback mechanism that inhibits food intake and increases energy consumption when body weight exceeds a certain value [42]. The inhibition is consequently removed when the body mass returns below this threshold. The hormones mainly involved in this regulation are leptin and ghrelin, which modulate the activity of neurons in the arcuate nucleus of the hypothalamus involved in the control of appetite [43]. In particular, the hypothalamus plays a central role in the regulation of food intake by means of the activity of the orexigenic (appetite-stimulating) neuropeptide Y (NPY) and agouti-related peptide (AgRP)-expressing AgRP/NPY neurons and the anorexigenic (appetite-suppressing) pro-opiomelanocortin (POMC)-expressing POMC neurons [44]. POMC neurons produce two different peptides involved in eating behavior, β-endorphins and melanocortins. The most important melanocortin is the α-melanocyte stimulating hormone (α-MSH), a potent appetite inhibitor [45].

Leptin is synthesized and secreted by adipose tissue in proportion to its mass. Once in circulation, leptin reaches the arcuate nucleus of the hypothalamus where it finds its JAK/STAT type receptors, mainly on two neuronal populations: NPY/AgRP neurons and POMC neurons. By inhibiting the former, and activating the latter, leptin is able to reduce the sense of hunger, decrease appetite, and increase basal metabolism. In particular, when fat mass decreases, plasma leptin levels fall: appetite is stimulated and energy expenditure is suppressed until the fat mass is restored. When fat mass increases, leptin levels increase and suppress appetite until weight is lost: the greater the fat mass, the greater its synthesis [46,47,48].

Obese subjects, although they have a high level of leptin in blood, exhibit leptin resistance which can be caused by (i) disorders of its transport across the blood brain barrier (BBB); (ii) the inhibition of leptin due to binding to circulating proteins, including plasma-soluble LepRb and C-reactive protein; (iii) changes to its receptor; and (iv) the overexpression of pro-inflammatory cytokines (e.g., SOCS3, PTP1B) inhibiting leptin signaling [49,50,51,52,53].

Ghrelin is a proteic hormone produced mainly by P/D1 cells lying at the bottom of the human stomach; is also expressed by the ε-cells of the pancreas and in an area of hypothalamus, namely, the arcuate nucleus. Ghrelin is a well-known hormone that stimulates food intake in a dose-dependent manner [54]. Blood levels of ghrelin increase before meals and decrease about an hour after food intake. It is therefore considered the complementary of the leptin hormone. Ghrelin increases appetite by stimulating the need for nutrition that is driven by metabolic needs but also by stimulating the need for nutrition induced by reward, memory, and motivated eating behavior, leading to deregulated weight gain and obesity [55]. Ghrelin receptors have the typical structure of G protein coupled receptors and are expressed by NPY neurons in the arcuate nucleus and the ventromedial hypothalamus. Ghrelin acts by activating NPY/AgRP neurons which, by releasing GABA into the synapse and inhibiting POMC (anorexigenic) neurons, prevent the production of α-MSH by the POMC neurons [56]. 

During a period of fasting, according to the energy needs of the organism, lipolysis is induced with the aim of releasing FFAs into circulation. The activation of lipolysis mainly depends on the action of hormone-sensitive lipase (HSL), an enzyme that hydrolyzes TGs releasing FFAs and glycerol [57]. FFAs are used by most tissues under conditions of prolonged hypoglycemia. The main lipolytic hormonal stimuli included leptin, catecholamines, glucagon, growth hormones (GH), cortisol, and thyroid-stimulating hormones (TSH) [58,59]. The activity of the enzyme is determined by its phosphorylation and the hormones that influence the lipolysis act by regulating this state. In particular, the phosphorylation and activation of HSL is induced by the cAMP-dependent protein kinase A (PKA) [60] and the AMP-activated protein kinase (AMPK) [61,62]. The most important anti-lipolytic hormone is insulin, whose action negatively regulates the phosphorylated state of HLS [63].

During a period of great energetic availability, the lipogenesis process is activated in AT. Lipogenesis in AT involves the re-esterification of FFAs derived by the hydrolysis of TGs endowed in lipoproteins VLDL and chylomicrons as well as *de novo* lipogenesis (DNL). The first process is regulated by the activity of the lipoprotein lipase (LPL), which acts by hydrolyzing the TGs of the lipoproteins, releasing FFAs and monoacylglycerols in adypocites [57]. After meals, insulin favors the expression of LPL in the vascular endothelium [64].

DNL is a highly regulated process in which carbohydrates from the circulation are converted into fatty acids which are then used to synthesize TGs or other lipid molecules [65]. Dysregulation of the DNL contributes to diseases such as obesity, type 2 diabetes, and cardiovascular diseases. The main transcription factor that regulates DNL at the adipose-tissue level is the carbohydrate response element binding protein nuclear transcription factor (ChREBP) [66,67], which is phosphorylated and inactivated by PKA and AMPK in conditions of energetic need. Other factors promoting DNL are the sterol regulatory element binding protein (SREBP) -1, liver X receptor (LXR) and polyunsaturated fatty acids [65,68,69].

### 3.2. Metabolic Syndrome (MS)

In recent years, obesity seems to have been classified among the main factors favoring the previously called Syndrome X, then Insulin Resistance Syndrome, and, more recently, metabolic syndrome (MS). Currently, the syndrome has been renamed “multimetabolic” because it encompasses a wide variety of pathophysiological problems including insulin resistance, altered glucose metabolism or diabetes mellitus, hypertriglyceridemia, and high levels of LDL cholesterol and low levels of HDL cholesterol, a condition that favors the onset of atherosclerotic plaques and arterial hypertension. MS is accompanied by a prothrombotic, inflammatory state and is associated with non-alcoholic fatty liver disease (NAFLD) [70,71,72].

The MS, according to the definition proposed by the WHO and then partially modified by the European Group for the Study of Insulin Resistance (EGIR), is characterized by insulin resistance, defined by the presence of hyperinsulinemia and high fasting blood glucose levels of at least 110 mg/dL, and the presence of at least two of the following criteria: (i) abdominal obesity defined on the basis of two definitions: according to the original WHO definition, it is a hip-waist ratio >0.90 or a BMI ≥30 kg/m^2^; according to the modification made by EGIR, it is instead the waist circumference ≥94 cm; (ii) dyslipidemia with serum triglycerides ≥150 mg/dL (≥1.70 mmol/L) or with HDL cholesterol ≤40 mg/dL (man) or ≤50 mg/dL (woman); and (iii) blood pressure >140/90 mmHg [73].

In a subject with MS, the excess energy is deposited in the form of TGs in existing adipocytes, causing significant hypertrophy; this determines the dysfunction of these adipocytes which carry out endocrine and immunological responses [74].

As previously mentioned, the mobilization of fat from adipocytes takes place by the hydrolysis of TGs deposited in the adipocytes and their subsequent release into the circulation of the hydrolysis products, e.g., FFAs or non-esterified fatty acids (NEFA). It has been observed that the visceral adipose tissue (VAT) of obese subjects is particularly sensitive to adrenergic stimulus mediated by the β3-receptor, which causes a marked lipolytic response [75,76,77]. In addition to the increased activity of the β3-adrenergic receptors, the increased lipolysis of visceral adipocytes depends on the reduced activity of the α2-adrenergic (antilipolytic receptors). The consequence of all of this is that in the portal circulation of obese subjects, there is a high release of FFAs in the bloodstream. It should be noted that VAT, the most pathogenic adipose tissue, is less susceptible to browning than subcutaneous adipose tissue (SAT) [78]. An anti-lipolytic action is given by insulin, whose receptors are poorly represented in the VAT. The insulin receptor is part of the large family of “tyrosine kinase” receptors, which is endowed with autocalatic kinase activity [79]; once the receptor is bound by insulin, it phosphorylates strategic tyrosine residues, resulting in the recall of a bank protein called insulin receptor substrate 1–4 (IRS 1–4), an activator of many protein-kinases that acts as a “system signal” [80]. In the obese subject, the mechanisms that regulate the activity of the insulin receptor are severely altered by factors that trigger the “switching off” reactions by the dephosphorylation of the receptor. In addition, phosphorylation on serine residues of IRS by serine/threonine kinases in obese subjects are responsible for insulin resistance [79,81]. Among the factors promoting insulin resistance are the high levels of circulating FFAs and the hypersecretion of cytokines as TNF-α [81,82]. In addition to inducing insulin resistance, circulating FFAs can activate pro-inflammatory pathways. In fact, it has been shown that (i) an excessive deposition of adipose tissue, especially in the visceral district, is characterized by an increased expression and release of pro-inflammatory cytokines such as tumor necrosis factor-α (TNF-α) and interleukin 6 (IL-6) and (ii) this excess of adipose tissue activates inflammatory signaling pathways as a result of the cellular dysregulation of homeostatic pathways, such as the stress response of the endoplasmic reticulum [83,84]. Furthermore, the release of FFA from these large adipocytes is able to activate the signaling of the Toll-like receptor (TLR) and, downstream, the Janus N-terminal kinase (JNK) and nuclear factor kB (NF-kB), signaling pathways in resident macrophages and inducing through these pathways a decisive change towards a classic pro-inflammatory phenotype. A direct consequence of this event is the increased production of cytokines, including IL-6 and TNFα, which interferes with the normal transmission of the insulin signaling favoring the onset of type 2 diabetes and further propagating the state of chronic inflammation [85,86].

## 4. Anti-Obesity Effect of EOs

Recent studies have shown that essential oils (EOs), thanks to their constituents, promote the reduction of fat mass and exert anti-obesity effects. It is important to note that EOs can exert these effects both when taken with the diet or inhaled [7,8,87]. 

In this section are described the main effects induced by EOs in reducing the metabolic effects that lead to obesity. We will first discuss the effects of EOs that act by inhalation. The volatile compounds present in EOs interact with specific olfactory receptors, stimulating the central nervous system (CNS) to regulate energy metabolism, regulating the balance between lipolysis and lipogenesis, through the regulation of appetite [88,89]. These effects are induced through the activation of the sympathetic and parasympathetic nervous systems and the release of hormones such as leptin and insulin [90,91]. 

Studies conducted in rats and mice have shown that olfactory stimulation with the scent of grapefruit EOs (*Citrus paradisi*, Pranarôm International, Belgium, GFO) increases the activity of the sympathetic nerves that innervate both white and brown adipose tissue, the adrenal glands and kidneys. This causes an increase in lipolysis, thermogenesis (with the consumption of fatty acids in the BAT), reduces appetite, and, consequently, body weight [88,92]. In addition, the GFO decreases the activity of the gastric vagal nerve [92,93]. Shen et al. [88] found that GFO, and in particular the limonene component, performs this effect through a histaminergic response. The participation of the complex histamine system in the regulation of body weight acts by mediating an action on dietary behaviors by activating the H3 receptors (presynaptic) to induce satiety [94] and collaborating with thyroid hormones in improving hyperlipidemia and its associated cardiovascular risk [95].

Furthermore, the inhalation of GFO decreases the activity of the vagal gastric nerve, reducing digestion and the absorption of nutrients with a possible decrease of appetite [88].

A similar effect was evidenced by the inhalation of citronella EOs (COE). In particular, Batubara et al. [96], using Sprague Dawley adult male rats as an experimental model, showed that the inhalation of COE extracted from *Cymbopogon nardus* L (Poaceae) Indonesian increased the activity of the sympathetic nervous system, decreasing the sense of appetite and consequently the body weight. The effect was mediated in particular by β-citronellol.

Particular interesting research have been performed with Patchouli EOs (PEO), a plant belonging to the lamiaceae family (*Pogostemon cablin Benth*), that have attracted the interest of many researchers for its anti-inflammatory, antiviral, antioxidant, and wound-repair properties [97,98]. Studies performed on male Sprague Dawley rats kept on a diet of high fat content (HFD) for 12 weeks demonstrated that the inhalation of PEO leads to a reduction in weight and serum leptin levels as well as decreases food intake [99]. The authors hypothesized that the reduction in leptin is due to a decrease in leptin resistance. The main components of PEO are citronellol, a volatile compound that fights obesity by reducing food intake, patchouli alcohol, α-patchoulene, and β-patchoulene, which stimulates the hypothalamus and regulate leptin levels [96,99].

In the pharmaceutical, food, and cosmetic fields, EOs extracted from oregano (EOO) are widely used. In fact, EOO, thanks to the presence of terpenes (both mono and sesquiterpenes), prove to possess great biological activity [100]. Among the main components of EOO, carvacrol, thymol, terpinen-4-ol, and linalool are of particular interest, which are present in different quantities depending on the species of oregano used (for example, H. patens, *L. grandis*, *O acutidens*, and *O vulgare*) [100,101,102]. Notably, several studies have highlighted the anti-obesity potential of oregano and, in particular, of the species with the highest carvacrol content. For example, it has been observed that carvacrol reduces lipid accumulation during adipogenic differentiation in human Wharton’s gelatin-derived mesenchymal stem cells (WJMSC) and murine 3T3-L1 cell lines. The effects seems to be ascribed to the modulation of genes linked to adipogenesis, such as transcription-factor ChREBP [16]. Moreover, it has been shown that carvacrol also reduces hypercholesterolemia and inflammation found in obese subjects. Cho et al. [103] demonstrated that carvacrol inhibits the expression of proteins associated with adipogenesis, such as SREBP-1, LXR, leptin, and LPL in male C57BL/6N mice.

In vitro studies have shown that EOO, particularly the extract from Origanum vulgare, inhibits lipogenesis in the human stomach cancer cell line (AGS). The authors highlighted that treatment with EOO reduces the expression of proteins involved in the biosynthesis of fatty acids and cholesterol, such as 3-hydroxy-3-methylglutaryl-coenzyme A reductase (HMGCR) and ACC [104].

The EOs extracted from sweet orange (*Citrus sinensis*, L; SOEO) have anti-obesity effects, highlighted with in vivo and in vitro experimental models. In particular it has been observed that SOEO, administered in microcapsules in obese SD rats, induces weight loss that is accompanied by a decreased expression of the receptor-γ activated by peroxisome proliferators (PPARγ) and ACC, which favors the lipogenesis of the subcutaneous adipose tissue as well as the up-regulation of UCP2, HSL, and carnitine palmitoyltransferase I, which favors the entry of fatty acids into the mitochondrion so that they are initiated for beta-oxidation [105]. Probably this ability is given by the presence of D-limonene, which has been demonstrated to inhibit adipocyte differentiation in adipocytes deriving from 3T3-L1 [106].

It has been shown that members of the transient receptor potential (TRP) superfamily have numerous biological functions, and TRP channels have become potential drug targets for a variety of pathophysiological conditions including obesity. Among the TRP channels, recent studies highlight in particular the role of the transient vanilloid receptor type 1 (TRPV1), the transient potential of the ankyrin receptor 1 (TRPA1), and the transient potential cation channel subfamily M (melastatin) member 8 (TRPM8) in the regulation of metabolism and energy homeostasis [107,108]. Many EOs, for example those extracted from bitter orange (*Citrus aurantiuum* L) and Spearmint *(Mentha spicata*), contain chemical compounds such as cinnamaldehyde, eugenol, and 1,8-cineole that, by stimulating these receptors, increase energy expenditure and thermogenesis and also reduce the sense of appetite and release of ghrelin [109,110,111,112].

OEs extracted from the leaves of cinnamon (*Cinnamomum osmophloeum* ct. linalool CiEO), a chemotype present in Taiwan, has a potential anti-obesity effect, probably determined by the constituent S—(+)—linalool [113,114]. Recent studies have shown that treatment with CiEO results in six-week-old male ICR mice weight loss and decreased blood triglyceride levels in male mice. Furthermore, treatment with CiEO inhibits lipid accumulation in 3T3-L1 adipocytes [113].

One strategy to reduce fat mass in the obese patient is to convert white adipocytes into brown-like adipocytes (beige or brite fat), a process called browning, which has the purpose of increasing energy expenditure by activating thermogenesis [115]. In fact, in beige adipocytes, as well as in brown ones, there is a greater expression of UCP1 which favors the production of heat as a form of energy thanks to the decoupling of the transport chain of electrons in the process of oxidative phosphorylation [116,117]. The EOs, thanks to their chemical components, can promote browning. The trans-anethole (trans-1-methoxy-4-propenyl-benzene) (TA) present in the EOs of various plants (eg fennel, anise, and star anise) has been shown to have anti-obesity properties favoring browning. Kang et al. [18] showed that the TA treatment of C57BL/6 mice induces the expression of beige adipocyte specific genes such as Ppargc1a, Prdm16, Ucp1, Cd137, Cited1, Tbx1, and Tmem26. Furthermore, TA showed thermogenic activity by increasing mitochondrial biogenesis in white adipocytes and activating brown adipocytes. In the experiments conducted, they also showed that TA reduces adipogenesis and lipogenesis and increases lipolysis and the oxidation of fats. The authors found that TA induces browning in 3T3-L1 adipocytes through the activation of the β3-adrenergic receptor and sirtuin1 (SIRT1). SIRT1 promotes the increase in the expression levels of proteins involved in lipid metabolism (UCP1, PRDM16, PGC-1a, AMPK, and pAMPK).

## 5. Effect of EOs on Metabolic Syndrome (MS) and Related Pathologies

Considering that obesity is among the conditions that can predispose one to the onset of metabolic syndrome (MS), EOs, due to their anti-obesity proprieties, can counteract its development.

Ginger (*Zingiber officinale*, Roscoe Zingiberaceae) is a medicinal plant used in the food field and as a natural remedy for the treatment of various gastrointestinal diseases (such as nausea, vomiting, and diarrhea) and for the treatment of cardiovascular diseases including arthritis, rheumatism, and muscle discomfort. The beneficial properties of ginger have been found in the root, where aromatic and pungent components, including essential oil and oleoresins, are present [118]. Ginger EOs (GiEO) are a mixture of monoterpenic and sesquiterpenic compounds that include zingiberene, β-bisabolene, γ-cadinene, β-sesquiphellandrene, neral, and geranial. Ginger oleoresin is a mixture of gingerols and shogaols, among which [6]-gingerol is a major pungent compound. Recent studies have shown that ginger has beneficial effects against metabolic disorders [119]. 

Studies conducted on male C57BL/6J mice subjected to a HFD have shown that the daily intake of GiEO has an anti-hyperlipedemic effect by reducing the serum levels of FFA, cholesterol, and triglycerides. Moreover, it was demondstrate that GiEO has antioxidant abilities and reduces inflammatory response in mouse livers, thus protecting steatohepatitis, a problem related to MS. These effects can be explained by the fact that GiEO induces a decrease in the levels of SREBP-1c, ACC, fatty acid synthase (FAS), HMGCR, and cytochrome P450 2E1 (CYP2E1). The observed effect is more evident if citral is added to the diet in addition to GiEO. Additionally, ginger extract has been shown to inhibit macrophage activation induced by LPS through the suppression of pro-inflammatory cytokines TNF-α [120,121].

EOs of *Salvia officinalis* L. (SEO) have been shown to have hypoglycemic and anti-obesity effects. In fact, the oral administration of SEO in male Wistar mice induced by alloxan has been shown to inhibit α-amylase and lipase and reduce glycemia and the level of glycogen stored in the liver. Furthermore, treatment with SEO has been shown to preserve hepatic functions, lowering the serum levels of AST, ALT, and LDH and renal activities, restoring serum concentrations of creatinine and uric acid [122,123]. It has also been shown that SEO can be useful as a dietary supplement in the prevention of type 2 diabetes mellitus by lowering the plasma glucose of individuals at risk [124].

Notably, SEO also has anti-tumor and antioxidant activities [125,126,127,128]. Furthermore, SEO treatment in hyperlipidemic mice on a high-fat diet reduced body weight gain, hyperlipidemia, and hypercholesterolemia and reduced the production of reactive oxygen species. The results of this research highlighted the beneficial effects of SEO in the management of these disorders without inducing side effects such as headache, constipation, and muscle pain. The results obtained show that SEO is more effective than simvastatin in improving the lipid profile and antioxidant activity, which could be due to the inhibition of dietary fat absorption and the regulation of fecal excretion [128].

Cumin EOs (CEO) is derived from *Cuminum cyminum*, a plant belonging to the *Apiaceae* family. In traditional medicine, CEO is used for its digestive and calming properties, and it has also been demonstrated to have anticancer properties. The main components present in CEOs are Cuminaldehyde (or 4-Isopropil Benzaldhyde), γ-terpinin, α-Sabinin, α a-Flandrene, and α -Kadinin. A study conducted in patients with type 2 diabetes found that consuming CEO causes a significant reduction in fasting blood glucose (FBS), glycosylated hemoglobin (HbA1c), and serum levels of insulin, adiponectin, and TNFα. These effects lead to a reduction in the inflammatory state [129]. Cuminaldehyde, which has an inhibitory effect on α-glycosidase and aldose reductase, two enzymes involved in carbohydrate metabolism, could give the anti-diabetic properties of the CEO. Furthermore, it was shown that in prediabetic patients, consuming CEO improved anthropometric indices (BMI and waist circumference (WC)), total serum cholesterol, and other markers of the lipid profile (LDL and HDL), especially in women, and contrasts insulin resistance. The authors, supported by the results obtained, conclude that CEO can be used as an adjuvant therapy for the metabolic state in pre-diabetics [130,131].

In general, it was demonstrated that the oral administration of cumin extract reduces the systolic blood pressure in hypertensive rats [132], but it also has beneficial effects on weight loss, hyperglycemia, and dyslipidemia in different diseases such as obesity, dyslipidemia, and type 2 diabetes [131,133].

Mosbah et al. pointed out that the essential oil of *Rhaponticum acaule* (L) DC (*R. acaule*; RaEO) consists of components with powerful antioxidant effects that can be used for therapeutic purposes. In particular, enzymatic kinetic studies have shown that RaEO has an inhibitory role against alpha glucosidase, xanthine oxidase, and pancreatic lipase. The inhibition of alpha glucosidase is one of the strategies used in diabetic patients [134].

In subjects with MS, there is hypertriglyceridemia and a high concentration of LDL cholesterol in the circulation [135]. Studies reported in literature show that *Melissa officinalis* EOs (MOEO) lowers plasma TG and cholesterol levels. In particular, it has been shown that in APOE2 transgenic mice and in lipid-loaded HepG2 cells, treatment with 400 and 800 mg/L of MOEO reduces the synthesis of fatty acids and cholesterol as a consequence of a decrease in the expression of factors related to their synthesis, such as SREBP-1c, ACC1, FAS, and SREBP-2 [136].

Yen et al. [137] evaluated the effect of twenty-nine commercial EOs (purchased at the Twain market) and, among them, three different MOEOs (distributed by companies) on the activity of metabolizing glucose (an in vitro antidiabetic screening model) and accumulating lipids on 3T3-L1 adipocytes. The authors found that MOEOE considerably increases glucose consumption and inhibits the accumulation of lipids in cells. This last effect was also induced by the EOs of peppermint, lavender, bergamot, cypress, niaouli nerolidol, rose geranium, and revensara. MOEO also determines the activation of AMPK, which favors the consumption of glucose, with the consequent inactivation of ACC, which inhibits the accumulation of lipids in adipocytes [137].

Talpur et al. evaluated the antidiabetic and antihypertensive potentials of three different formulations of EOs: EO1 (pumpkin seed oil, extra virgin olive oil, oregano, cinnamon, fenugreek, cumin, and fennel), EO2 (pumpkin seed oil, extra virgin olive oil, oregano, cinnamon, fenugreek, cumin, blueberry, allspice, and ginger), and EO3 (pumpkin seed oil, extra virgin olive oil, oregano, cinnamon, fenugreek, cumin, and blueberry). The evaluation of the effects of the three compositions of EOs was conducted on Zucker fatty rats (ZFR), a model of obesity and insulin resistance, and on spontaneously hypertensive rats (SHR), a model of genetic hypertension. The experiments conducted showed that all three combinations of EOs, and in particular EO3, lower blood glucose levels and systolic blood pressure in both ZFRs and SHRs [138]. 

The effects highlighted suggest that EOs may increase insulin sensitivity. In fact, many studies show that the EOs of fenugreek and cinnamon possess antidiabetic properties; in particular, the EOs of fenugreek block the absorption of glucose while cinnamon mimics the action of insulin [139,140,141,142].

Mitochondria in adipose tissue produce large amounts of ROS, their dysfunction causing increased oxidative stress and inflammation which may be the link between obesity and associated cardiovascular and metabolic complications [143].

EOs extract from *Campomanesia phaea (O.Berg) Landru* (CpEO) leaves have been shown to exert antioxidant and anti-inflammatory effects. In particular, it has been demonstrated that treatment with CpEO decreases the production of proinflammatory mediators (IL-6 and TNF-α), NO, and O_2_^−^ induced by LPS in RAW 264.7 macrophages. Such anti-inflammatory effects can be explained by the inhibition of the NF-kB signaling pathway [144]. In addition, the polyphenols of the fruit of *Campomanesia phaea* (O. Berg.) have a therapeutic action that improves the complications associated with obesity, such as inflammation, hepatic steatosis, hyperglycemia, glucose intolerance, and insulin resistance, by the activation of Akt and AMPK [145].

Studies conducted on the EOs of garlic (GEO) and its main organosulfur component (diallyl disulfide, DADS) highlighted their great anti-inflammatory power. Treatment with GEO reduced the release of pro-inflammatory cytokines in the livers of C57BL/6 mice accompanied by a high antioxidant capacity through the inhibition of cytochrome P450 2E1 expression. Treatment with GEO decreased the development of non-alcoholic fatty liver disease (NAFLD), anti-obesity, and antihyperlipidemic effects and also reduced the body weight of mice. The anti-NAFLD effects of GEO are mediated by the downregulation of SREBP-1c and ACC and by the activation of PPARα, which induces hepatic lipolysis. Similar effects have been highlighted by treating mice with DADS, which suggests that this is an active chemical component that gives GEO anti-inflammatory and anti-obesity properties [146].

Hyperglycemia, present in the diabetic or prediabetic subject, causes chronic inflammation and contributes to an increase in the production of reactive oxygen species (ROS), which in turn is responsible for vascular dysfunction [147]. 

*Zataria multiflora* (Shirazi thyme) is a medicinal plant belonging to the Lamiaceae family with antioxidant and anticancer properties [148,149]. The EOs extracted from Zataria multiflora (ZMEO), whose main components are phenolic monoterpenoids (thymol and carvacrol), monoterpenes (para-cymene and gamma-terpinene), alcoholic monoterpenoids (linalool), sesquiterpenes (caryophyllene and cadinene), and sesquiterpenoids (spatulenol), have a high antioxidant power that can be used in the antioxidant therapy of diabetes [149]. Aminizadeh et al. evaluated the ZMEO effects, administered via dendrosome, against oxidative stress induced by hyperglycemia in the hematopoietic cell line of mouse macrophages (J774 A.1). Studies have shown that ZMEO reduces the levels of oxidative stress markers such as NOX, Nrf2, NF-kB, and the levels of intracellular hydrogen peroxide while increasing the expression and activity of superoxide dismutase (SOD) and catalase, thereby reducing the lipid oxidation, oxidation, and glycation of proteins [150].

## 6. Effect of EOs on Microbiota

It is interesting to highlight the effect of EOs on the microbiota, which play an important role in predisposing and promoting the onset of obesity.

The intestinal microbiota is a microbial community of the enteric tract, consisting mainly of bacteria as well as yeasts, parasites, and viruses [151]. The intestinal microbiota must be considered a real organ that communicates with the host. It plays different roles in host health by preserving an intestinal barrier against hexogen microbes, stimulating the immune system, metabolizing dietary nutrients and drugs, and synthesizing vitamins and bioactive molecules [152,153]. Furthermore, the components of the microbiota may enter the circulation and be transported to various organs (brain, liver, pancreas, adipose tissue, etc.) affecting their functionality [152].

The composition of the gut microbiota is strongly influenced by different factors such as the microbial species acquired at birth, host genetics, immunological factors, antibiotic usage, and health status. However, diet is considered among the most crucial factors affecting microbiota composition [151,154]. A gut microbiota in a eubiotic status is characterized by a preponderance of potentially beneficial species belonging mainly to the two bacterial phylum *Firmicutes* and *Bacteroides*, while potentially pathogenic species, such as those belonging to the phyla *Proteobacteria* (*Enterobacteriaceae*), are present but in a very low percentage. A change in the ratio between “good bacteria” and “bad bacteria” is referred to as dysbiosis, with consequences in host health.

Emerging evidence suggests a causal link between microbial dysbiosis and obesity [11]. Studies conducted in both mice and humans have shown a change in the composition of the intestinal microbiota in obese subjects, with an increase in Firmicutes and a reduction in *Bacteroidetes* [154,155]. This has been correlated with a high fat diet of obese subjects [11]. A change in microbiota composition in obese subjects in turn affect the body’s nutritional and metabolic balance by modulating its ability to extract energy from dietary foods and interacting with its glyco–lipid metabolism. The metabolites released by the fermentation of complex polysaccharides of the diet can increase glucose absorption, stimulate lipogenesis, and modify the fatty acid composition of adipose tissue and liver, thus favoring fat mass increase. Furthermore, dysbiosis associated with obese subjects has been reported to alter the permeability of the intestinal mucosal barrier and immune response, contributing to a state of chronic systemic inflammation and favoring insulin resistance [9,10]. The link between the bacteria in our microbiota and weight gain/loss is a quite an active research field.

Several recent studies have sustained the value of EOs added to the diet as components that can affect the composition of the microbiota. It has been shown that the intake of microcapsules containing EOs of sweet orange (*Citrus sinensis* L. *Osbeck*; SOEO) increase the protection of the gut barrier in obese rats and the reduction of endotoxins, with variation in the composition of the microbiota by increasing *Bifidobacterium*. In addition, SOEO microcapsules promote weight loss in mice [156]. Wang et al. demonstrated that in male mice the intragastric administration EOs of orange, limonene, linalool, and citral influence the intestinal microbiota, increasing the quantity of Lactobacillus and significantly reducing the content of short-chain fatty acids in the cecum and the colon [157].

A high-fat diet increases the levels of both plasma and fecal endotoxins, proinflammatory cytokines, the induction of the TLR4, iNOS, and COX-2, and the activation of NF-κB in the colon and also causes the dysregulation of gut microbiota by increasing the Firmicutes/Bacteriodetes ratio [158]. Cinnamon EO (CiEO) administration is effective in preventing inflammation induced by dextran sodium sulfate (DSS) and in modifying gut microbial dysbiosis. In particular, it promotes the increase of probiotic intestinal bacteria such as Bacteroidales S24-7, bacteria (*Alloprevotella* and *Lachnospiraceae_NK4A136*) that produce short-chain fatty acids (SCFA) and a decrease in Helicobacter and Bacteroides, correlated with the increase in TLR4 and TNF-α [159].

Studies conducted by Leong et al. have shown that PEO possesses a prebiotic-like effect in C57BL/6J mice, (i) restoring the expressions of E-cadherin and N-cadherin, (ii) increasing the expression of the p-lysozyme and Muc 2 genes, which are important for the functionality of the intestinal barrier, and (iii) suppressing the expression of pro-inflammatory cytokines. In addition, the intake of PEO favors the production of SCFA by increasing bacteria in the intestine, e.g., *Anaerostipes butyraticus*, *Butytivibrio fibrisolvens*, *Clostridium jejuense*, *Eubacterium uniform* and *Lactobacillus lactis*, while reducing the presence of pathogens such as *Sutterella* spp., *Fusobacterium mortiferum*, and *Helicobacter* spp. [160].

## 7. Conclusions

In conclusion, thanks to the presence of various chemical constituents, EOs show various beneficial properties for health (Table 1). Several in vivo and in vitro studies have shown that they have anti-obesity effects by modulating lipolysis and lipogenesis, stimulating browning, and varying leptin levels. In addition to these effects, the use of EOs or some of their chemical constituents such as carvacrol, trans-anethole, or limonene can counteract the consequences induced by the increases in fat mass in obese patients, such as type 2 diabetes, hypertension, and cardiovascular risks. It should be noted that EOs can influence the composition of the intestinal microbiome, in favor of Bifidobacterium for example, thus reducing the physiological risk of metabolic syndrome in overweight subjects. 

The studies reported in this review highlight that there are many experimental data indicating the potential of EOs in the prevention and/or treatment of obesity and related diseases. These provide a good starting point for investigating the effects of EOs in clinical studies.

The beneficial effects of EO intake, through diet or inhalation, can be potentiated in a subject who follows a correct diet and constant physical activity and avoids a sedentary life.

## Figures and Tables

**Table 1:** This table shows the main effects of the EOs described in the review with the relative references (↓ decrease effect/factor; ↑ increase effect/factor).

	Effect of Essential Oils (Eos) on:				
EOs	Obesity	Metabolic Syndrome and Related Pathologies	Microbiota	Model System	Ref
Campomanesia EOs (CpEO)		Antioxidant and anti-inflammatory effects; ↓insulin resistance and hepatic steatosis		Obese C57BL/6J mice; RAW 264.7 macrophages	[145,146]
Cinnamon EOs (CiEO)	↓body weight	↓hyperlipidemia	Probiotic effect; ↓Helicobacter and Bacteroides	Six-week-old male ICR mice; APOE2 transgenic mice	[113,114,159]
Citronella EOs (COE)	↓appetite and body weight			Sprague Dawley adult male rats	[96]
Cumin EOs (CEO)	↓body weight	↓fasting blood glucose, glycosilated hemoglobin, insulin, inflammation, hypertension		In vivo models (diabetic patients); hypertensive rats	[129,130,131]
Garlic EOs (GEO)	↑lipolysis	↓NAFLD		C57BL/6 mice	[146]
Ginger EOs (GiEO)		↓FFA and inflammation		Obese mice	[120,121]
Grapefruit EOs (GFO)	↑lipolysis and thermogenesis; ↓appetite	↓hyperlipidemia and cardiovascular risk		Rats and mice	[88,92,93,94,95]
Melissa officinalis EOs MOEO		↓ plasma TG and cholesterol levels		APOE2 transgenic mice; APOE2 transgenic mice	[136,137]
Oregano EOs (EOO)	↓adipogenesis	↓hypercholesterolemia and inflammation		Cell lines; C57BL/6 mice	[16,100,101,102,103]
Patchouli Eos (PEO)	↓leptin resistance and food intake		Prebiotic effect	Sprague Dawley male rats; C57BL/6 mice	[97,98,99,160]
Rhaponticum acaule EOs (RaEO)		Antioxidant; antidiabetic		Enzyme kinetic studies	[134]
Salvia EOs (SEO)	↓body weight	↓hyperglycemia and hyperlipipidemia; antioxidant effects		Hyperlipidemic mice; diabetic rats; in vivo models (diabetic patients)	[122,123,124,129]
Sweet Orange EOs (SOEO)	↑lipolysis; ↓lipogenesis and body weight	↓dysplipidemia and hyperglycemia	↑protection of gut barrier; ↑Bifidobacterium and Lactobacillus; ↓endotoxin	Obese SD rats; mice	[105,106,156,157]
Zataria multiflora EOs (ZMEO)		Antioxidant		Mouse macrophages (J774 A.1)	[149,150]

## Data Availability

Not applicable.

## Abbreviations

ACC	acetyl-CoenzymeA carboxylase
AgRP	agouti-related peptide
AMPK	AMP-activated protein kinase
AT	adipose tissue
BAT	brown adipose tissue
BBB	blood brain barrier
BMI	Body Mass Index
CEO	cumin essential oils
ChREBP	carbohydrate response element binding protein nuclear transcription factor
CNS	central nervous system
COE	citronella essential oils
CiEO	cinnamon essential oils
CpEO	Campomanesia phaea (O.Berg) Landru essential oils
CYP2E1	cytochrome P450 2E1
DNL	de novo lipogenesis
EGIR	european group for study of insulin resistance
EOO	oregano essential oils
EOs	essential oils
FAs	fatty acids
FAS	fatty acid synthase
FBS	fasting blood glucose
FFA	free fatty acids
GEO	garlic essential oils
GFO	grapefruit essential oil
GH	growth hormone
GiEO	ginger essential oils
HbA1c	glycosylated hemoglobin
HFD	high fat content
HMGCR	3-hydroxy-3-methylglutaryl-coenzyme A reductase
HSL	hormone-sensitive lipase
IL- 6	interleukin 6
IRS 1–4	insulin receptor substrate 1–4
JNK	Janus *N*-terminal kinase
LDs	lipid droplets
LPL	lipoprotein lipase
LPS	lipopolysaccharides
LXR	liver X receptors
MAPK	mitogen-activated protein kinase
MOEO	melissa officinalis essential oils
MS	metabolic syndrome
NAFLD	non-alcoholic fatty liver disease
NEFA	non esterified fatty acids
NF-kB	nuclear factor kB
NPY	neuropeptide Y
PAI-1	plasminogen activator inhibitor-1
PEO	patchouli essential oils
PKA	cAMP-dependent protein kinase A
POMC	pro-opiomelanocortin
PPAR γ	receptor-γ activated by peroxisome proliferators
RaEo	Rhaponticum acaule essential oils
ROS	reactive oxygen species
SAT	subcutaneous adipose tissue
SCFA	short-chain fatty acids
SEO	salvia officinalis essential oils
SHR	spontaneously hypertensive rats
SIRT1	sirtuin1
SOD	superoxide dismutase
SOEO	sweet orange essential oils
SREBP	sterol regulatory element binding protein
TGs	triacylglycerols
TLR	toll-like receptor
TNFα	tumor necrosis factor α
TRP	transient receptor potential
TRPA1	transient potential of the ankyrin receptor 1
TRPM8	transient potential cation channel subfamily M (melastatin) member 8
TRPV1	transient vanilloid receptor type 1
TSH	thyroid-stimulating hormone
UCP-1	uncoupling protein-1
VAT	visceral fat adipocytes
WAT	white adipose tissue
WC	waist circumference
WHO	world health organization
WJMSC	Wharton’s gelatin derived mesenchymal stem cells
ZFR	Zucker fatty rats
ZMEO	Zataria multiflora essential oils
α-MSH	α-melanocyte stimulating hormone
